# Decreased levels of serum cytokeratin 19 fragment CYFRA 21-1 predict objective response to chemotherapy in patients with non-small cell lung cancer

**DOI:** 10.3892/etm.2013.1171

**Published:** 2013-06-20

**Authors:** LI PANG, JING WANG, YANWEN JIANG, LIANGAN CHEN

**Affiliations:** 1 Department of Respiratory Medicine, Chinese PLA General Hospital, Beijing 100853;; 2 Department of Pulmonary and Critical Care Medicine, Beijing Shijitan Hospital, Beijing 100050, P.R. China

**Keywords:** cytokeratin 19 fragment CYFRA 21-1, chemotherapy, non-small cell lung cancer, receiver operating characteristic analysis

## Abstract

Diagnostic tools capable of predicting early responses to chemotherapy are required to improve the individual management of cancer patients. The present study aimed to evaluate the prognostic significance of the serum tumor markers CYFRA 21-1, carcinoembryonic antigen (CEA), neuron-specific enolase (NSE), carbohydrate antigen (CA) 125, and CA 19-9 for predicting responses to different chemotherapy regimens in patients with non-small cell lung cancer (NSCLC). A total of 276 patients with postoperative stage I–IV NSCLC were retrospectively reviewed. The five tumor markers were measured before and after at least two cycles of chemotherapy using an electrochemiluminescent assay. Multivariate analysis revealed that performance status, age, postoperative stage and surgery were significantly associated with the response to chemotherapy. High baseline CYFRA 21-1 and CA 19-9 levels were associated with poor effectiveness of chemotherapy. Significant reductions in CYFRA 21-1 levels were associated with a positive response to various chemotherapy regimens. CEA, CA 125 and CA 19-9 expression was only associated with a positive response in patients receiving paclitaxel, docetaxel, pemetrexed and the epidermal growth factor receptor (EGFR) tyrosine kinase inhibitor (TKI). NSE expression was only associated with a positive response to gemcitabine. Receiver operating characteristic (ROC) curve analysis indicated that CYFRA 21-1 is the most sensitive of the tumor markers in predicting the response to chemotherapy. Serum CYFRA 21-1 is a useful surrogate marker for predicting the response to different chemotherapy regimens used to treat NSCLC and is a more sensitive marker than CEA, CA125, CA19-9 and NSE.

## Introduction

Lung cancer is one of the leading causes of morbidity and mortality worldwide (1). Non-small cell lung cancer (NSCLC) accounts for >80% of total lung cancer cases. NSCLC may be removed surgically if diagnosed in the early stages (2). However, the majority of patients are diagnosed at more advanced stages of the disease when surgery is no longer possible. Such patients are candidates for chemotherapy, which is associated with high toxicity and high cost, with limited efficacy.

Patients undergoing chemotherapy require monitoring to assess tumor progression. Various techniques, including computed tomography (CT) and positron-emission tomography (PET), alone and in combination with specific biomarkers are routinely used (3). Compared with other methods, tumor markers provide an economical and convenient method for detecting early tumor recurrence (4,5). Several markers have been shown to be sensitive and effective in monitoring NSCLC (5,6). However, due to their lack of specificity, the identification of new markers for NSCLC progression in patients undergoing different chemotherapy regimens is required.

Cytokeratin 19 (CK 19) is a constituent of the intermediate filament protein responsible for the structural integrity of epithelial cells. It has been used as a surrogate biomarker of circulating tumor cells of epithelial origin, including adenocarcinoma. A fragment of CK 19, CYFRA 21-1, has been proposed as a tumor marker for several malignancies, including NSCLC (5,7).

Carcinoembryonic antigen (CEA) is a cell surface anchoring protein. It is a member of the immunoglobulin cell adhesion molecule (IgCAM) superfamily and participates in cell adhesion. High serum concentrations of CEA have been shown to be associated with advanced disease and tumor relapse in patients with NSCLC and other cancers (8,9).

The neuroendocrine marker neuron-specific enolase (NSE) is a glycolytic enzyme associated with high diagnostic sensitivity and specificity in small cell lung cancer (SCLC); however, its use in monitoring the effect of chemotherapy in NSCLC is controversial (10,11).

The aim of the present study was to evaluate the prognostic significance of serum tumor markers in predicting the response to different chemotherapy regimens in patients with NSCLC.

## Patients and methods

### Patients

Data from 276 patients diagnosed with NSCLC at the Department of Pulmonary and Critical Care Medicine, Beijing Shijitan Hospital, Capital Medical University (Beijing, China) between February 2009 and October 2012 were retrospectively reviewed. All patients had a recent histological diagnosis of NSCLC classified according to the World Health Organization criteria (12). None of the patients had received previous treatment for NSCLC and none had a history of other forms of malignancies. Clinical data were recorded, including age, gender, disease stage and Eastern Cooperative Oncologic Group (ECOG) performance status.

The majority of the patients (180 patients, 65.22%) received a four-cycle chemotherapy regimen or 4 months of treatment with an epidermal growth factor receptor (EGFR) tyrosine kinase inhibitor (TKI). A number of patients (63 patients, 22.83%) received no further treatment. However, 33 patients (11.96%), who were in disease progression, only received two cycles of chemotherapy or 2 months of treatment with an EGFR kinase inhibitor.

Chemotherapy included third-generation platinum-based regimens used in combination with gemcitabine, paclitaxel, docetaxel or pemetrexed. Response, based on CT scans performed every 2 months after treatment, was evaluated according to the Response Evaluation Criteria in Solid Tumors (RECIST) criteria (13). Outcomes were classified as complete response (CR), partial response (PR), progressive disease (PD) or no change (NC). Written informed content was obtained from the patients. The study was approved by the Ethics Committee of Beijing Shijitan Hospital, Capital Medical University.

### Blood samples

Blood samples were collected within 14 days before surgery or chemotherapy and 3 weeks after completion of the second or fourth chemotherapy cycles. On the two occasions, CYFRA 21-1, CEA, NSE, CA 125 and CA 19-9 were measured by automated electrochemiluminescent assays (Roche Diagnostics GmbH, Mannheim, Germany).

### Statistical analysis

Statistical analysis was performed using SPSS version 16.0 (SPSS, Inc., Chicago, IL, USA). χ^2^ and logistic regression analyses were used to evaluate associations between tumor response and baseline clinical variables. Independent sample t-tests were used to analyze correlations between outcome and blood levels of CEA, CYFRA 21-1, CA 125, CA 19-9 and NSE. Numerical values for the tumor markers were transformed using natural logarithms to obtain near normal data distribution. Receiver operating characteristic (ROC) curves were created to evaluate the role of different tumor markers in predicting the response to chemotherapy. Cut-off values were determined by the Youden’s index and the area under the curve was used as a measure of sensitivity. P<0.05 was considered to indicate a statistically significant difference.

## Results

### Decline in CYFRA 21-1 predicts response for different chemotherapy in NSCLC

The baseline characteristics of the patients included in the study are shown in Table I. The study included 172 males and 104 females with a median age of 63 years (range, 29–88 years). The majority of tumors were adenocarcinoma (70.7%); squamous cell carcinoma (SCC) accounted for 26.1% of tumors and other histological types for 3.2%. The majority of patients (51.8%) received surgery, with the exception of those diagnosed as clinical Stage IV.

The treatment response was CR in 64 patients, PR in 47 patients, NC in 69 patients and PD in 96 patients. Based on the different treatment options, patients were divided into five groups as follows: a surgery only group (25 SCC and 38 adenocarcinoma patients); a paclitaxel or docetaxel (TP) group (19 SCC and 54 adenocarcinoma patients); a gemcitabine (GP) group (24 SCC and 30 adenocarcinoma patients); a pemetrexed (PP) group (0 SCC and 47 adenocarcinoma patients) and a TKI group (0 SCC and 33 adenocarcinoma patients). Thirty-two samples (11.6%) were collected following the second cycle of treatment in cases where the patients were unable to tolerate further chemotherapy or their response was evaluated as PD.

Multivariate analysis revealed that performance status, age, postoperative stage and surgery were significantly correlated with the response to chemotherapy (Table II). However, there was no significant association between tumor response and gender, tumor type, chemotherapy regimen or baseline serum levels of tumor markers.

Patients were further divided into high and low tumor marker groups based on the pretreatment marker levels. The cut-off values that defined these groups are shown in Table III. Correlation analysis revealed that baseline CYFRA 21-1 (P= 0.047) and CA 19-9 (P<0.001) were significantly correlated with effectiveness of chemotherapy (Table III). No similar associations were identified for CEA, CA 19-9 and NSE (Table III).

Table IV shows the correlations between changes in tumor markers following chemotherapy and the effectiveness of different chemotherapy regimens. Reductions in the serum levels of CYFRA 21-1, CEA, CA 19-9 and CA 125 were significantly associated with outcome in the surgery only group. Reduced serum levels of CEA, CYFRA 21-1 and CA 19-9 were significantly correlated with effectiveness of TP and PP chemotherapy. CYFRA 21-1 and NSE were significantly associated with responses to gemcitabine. Responses in the TKI group were associated with reductions in the serum levels of CEA, CYFRA 21-1 and CA 125.

**Figure 1.**
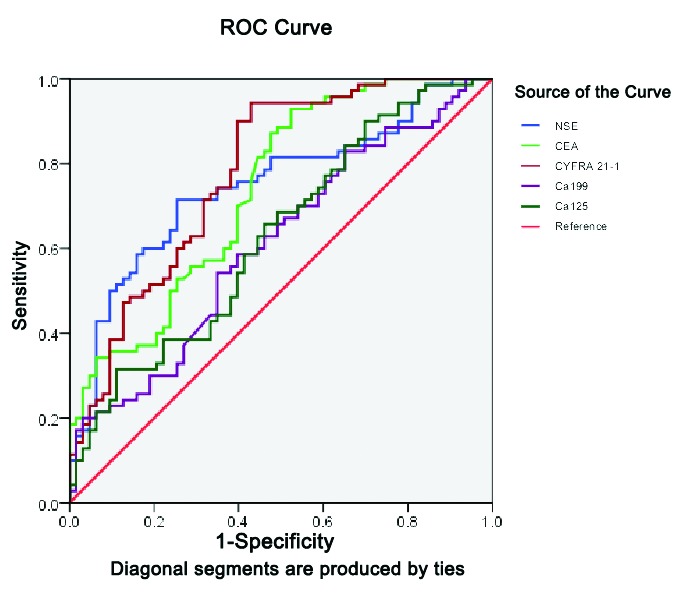
Receiver operating characteristic (ROC) curves for the responses of tumor markers in relation to the effectiveness of chemotherapy. NSE, neuron-specific enolase; CEA carcinoembryonic antigen; CYFRA 21-1, cytokeratin 19 fragment; CA, carbohydrate antigen.

The reductions of tumor markers in relation to the effectiveness of chemotherapy are shown in Fig. 1 and Table V. Among all relevant markers, CYFRA 21-1 yielded the most promising sensitivity at 90%. The corresponding area under the ROC curve was 0.779 (95% CI, 0.700–0.858; P<0001).

## Discussion

Chemotherapy is one of the main methods of treatment for NSCLC. Efficacy is routinely evaluated on the basis of radiological findings; however, this is not conducive to the early detection of recurrence and metastasis. Consequently, there is growing demand for convenient tools for estimating prognosis and for detecting responsiveness to therapy in order to optimize disease management on an individual basis. Several tumor markers, including CEA and CYFRA 21-1 in NSCLC, have been previously shown to provide useful estimates of prognosis (14–16). However, their role in the evaluation of treatment effectiveness is controversial, partly due to the lack of comparative studies evaluating different chemotherapy regimens.

It is well recognized that baseline parameters, including histology, gender, World Health Organization performance status, number of positive lymph nodes, gross tumor volume and high levels of tumor markers are associated with poor chemotherapy efficacy and poor survival (5,6,17). In the present study we identified significant associations between performance status, age, postoperative stage and chemotherapy response; however, we did not identify similar associations for gender, tumor type, chemotherapy regimen or baseline tumor markers.

CYFRA 21-1 is a fragment of CK 19, mainly present in the cytoplasm of tumor cells of epithelial origin, including lung and esophageal cancer. Expression of CYFRA 21-1 has been detected in lung adenocarcinoma and SCC (15). CYFRA 21-1 has also been identified as a potential tumor marker for the diagnosis and prognosis of NSCLC. CYFRA 21-1 has previously been identified as a valuable marker for the individual management of patients with recurrent NSCLC receiving second-line chemotherapy (18). In the present study we demonstrated that high serum CYFRA 21-1 levels are indicative of chemotherapeutic effectiveness in NSCLC. This observation is consistent with a previous study reporting that baseline serum levels and changes in CYFRA 21-1 are reliable markers for chemotherapy response in NSCLC (15).

In the present study patients were analyzed in five groups according to the type of chemotherapy received. The majority of patients in the TP and GP groups had NSCLC, while all patients in the PP and TKI groups had adenocarcinoma. Comparison of the changes in serum tumor markers in the different groups revealed that reductions in CYFRA 21-1 were significantly associated with a positive response to chemotherapy in all groups. This finding may be related to the fact that CYFRA 21-1 expression is dependent on tumor stage as opposed to histological type (7,18–20). Two previous studies (21,22) demonstrated that CYFRA 21-1 is an independent prognostic factor in the earlier stages of SCC. However, data from another study (5) suggest that CYFRA 21-1 may be a reliable surrogate marker of chemotherapy efficacy in patients with advanced NSCLC.

CYFRA 21-1 is ubiquitously found in a number of body tissues and has been described as a marker of apoptotic cell death (23). This suggests that CYFRA 21-1 may be a tumor marker in SCLC as well as in NSCLC. One study demonstrated that high levels and insufficient reductions of CYFRA 21-1 during the first and second cycles of chemotherapy were correlated with poor outcome in 128 patients with newly diagnosed SCLC receiving first-line chemotherapy (19).

CEA is widely used as a tumor marker in lung, gastrointestinal and gynecological cancers, as well as other tumors. The majority of patients with NSCLC who have elevated CEA levels have adenocarcinoma (23–26). CEA is a member of the IgCAM superfamily and is involved in cell adhesion and transfer (27,28). High serum levels of CEA have been shown to be a risk factor for the development of brain metastasis and are associated with poor prognosis (9). We identified no significant association between high baseline serum CEA levels and a poor response to chemotherapy. However, there was a significant association between decreased serum CEA levels and the effectiveness of chemotherapy in the groups where the majority of patients had adenocarcinoma. CEA may, therefore, represent a molecular target for lung adenocarcinoma. Similar results were observed for reductions in serum CA 125 and CA 19-9. All three markers are derived from glandular cells, which may make them suitable for monitoring the effectiveness of treatment in lung adenocarcinoma (29–31). However, these three markers are non-specific and it may be necessary to combine results for other biomarkers in order to increase sensitivity and specificity (6,32,33).

Changes in serum NSE predicted responses only in the GP group. This may be explained by the fact that the majority of patients receiving this form of chemotherapy had SCC. Previous studies have identified NSE as a sensitive tumor marker for SCLC (10), but not for NSCLC (20,34). More recent results are contradictory. One study concluded that patients without elevated NSE mRNA had a better prognosis compared with patients who experienced an increase in NSE, while another study concluded that pretreatment NSE mRNA had potential as a prognostic biomarker for advanced NSCLC (20,35).

Published evidence indicates that the most convincing data available among potential tumor markers for NSCLC, is that obtained with CYFRA 21-1 (15,18,36,37). In the present study, CYFRA 21-1 yielded the most promising sensitivity (90%) and the largest area under the ROC curve, compared with the other tumor markers.

This retrospective investigation of prognostic tumor markers suggests that changes in serum levels of CYFRA 21-1 may be helpful in predicting the effectiveness of different chemotherapy regimens used to treat NSCLC. We also identified serum CEA, CA 125 and CA 19-9 as possible prognostic factors in lung adenocarcinoma.

## Figures and Tables

**Table I.** Baseline characteristics.

Variable	N	(%)
Gender		
Male	172	62.3
Female	104	37.7
ECOG PS score		
≤1	233	84.4
>1	43	15.6
Histological type		
Squamous cell carcinoma	72	26.1
Other	204	73.9
Postoperative stage		
I	34	12.6
II	44	15.9
IIIa	27	9.8
IIIb	28	10.1
IV	143	51.8
Surgery		
Yes	143	51.8
No	133	48.2
First line treatment		
NO	63	22.8
TP	73	26.4
GP	54	19.6
PP	47	17.0
TKI	33	12.0
Other	6	2.2

ECOG, Eastern Cooperative Oncology Group; PS, performance status; NO, surgery only; TP, paclitaxel or docetaxel; GP, gemcitabine; PP, pemetrexed; TKI, tyrosine kinase inhibitor.

**Table II.** Logistic regression analysis of the correlation between effectiveness of chemotherapy and clinical factors.

Variable	Odds ratio	95% CI		P-value

		Lower	Upper	
ECOG PS score	2.225	1.073	4.616	0.032^a^
Age	1.053	1.009	1.099	0.018^a^
Gender	0.759	0.353	1.631	0.480
Histological type	0.977	0.422	2.263	0.957
Stage	2.366	1.038	5.395	0.041^a^
Surgery	3.441	1.431	8.273	0.006^a^
Chemotherapy regimen	1.033	0.759	1.407	0.837
CEA	1.000	0.997	1.002	0.679
CYFRA 21-1	1.010	0.983	1.038	0.465
CA 125	0.999	0.997	1.002	0.327

a P<0.05. ECOG, Eastern Cooperative Oncology Group; PS, performance status; CEA, carcinoembryonic antigen; CYFRA 21-1, cytokeratin 19 fragment; CA 125; carbohydrate antigen 125.

**Table III.** Correlation between effectiveness of chemotherapy and baseline serum levels of tumor markers.

Variables	N	CR + PR	NC + PD	χ^2^	P-value
CYFRA 21-1 (ng/ml)					
<3.3	98	53	45		
≥3.3	130	50	80	3.935	0.047^a^
CEA (ng/ml)					
<5.0	143	63	80		
≥5.0	129	47	82	1.636	0.201
CA 125 (U/ml)					
<35	135	69	66		
≥35	107	34	73	0.710	0.791
CA 19-9 (U/ml)					
<37	210	81	119		
≥37	42	12	30	21.885	<0.001^a^
NSE (ng/ml)					
<17	145	70	75		
≥17	68	25	43	2.482	0.115

a P<0.05. CYFRA 21-1, cytokeratin 19 fragment; CEA, carcinoembryonic antigen; CA, carbohydrate antigen; NSE, neuron-specific enolase; CR, complete response; PR, partial response; NC, no change; PD, progressive disease.

**Table IV.** Correlation between responses of tumor markers and effectiveness of different chemotherapy regimens.

Regimen	CR + PR	NC + PD	t-value	P-value
NO				
CEA	25	36	3.953	<0.001^a^
CYFRA 21-1	17	29	2.838	0.007^a^
CA 19-9	21	25	2.178	0.039^a^
CA 125	21	30	2.361	0.025^a^
NSE	19	22	1.303	0.200
TP				
CEA	34	39	4.889	<0.001^a^
CYFRA 21-1	32	30	3.975	<0.001^a^
CA 19-9	24	26	2.290	0.026^a^
CA 125	32	32	1.952	0.059
NSE	23	21	1.571	0.124
GP				
CEA	25	27	1.912	0.062
CYFRA 21-1	22	20	2.419	0.020^a^
CA 19-9	21	22	1.004	0.327
CA 125	22	25	0.968	0.338
NSE	20	18	3.003	0.004^a^
PP				
CEA	21	26	3.813	<0.001^a^
CYFR 21-1	20	17	2.715	0.010^a^
CA 19-9	18	20	2.563	0.015^a^
CA 125	10	19	1.201	0.238
NSE	16	12	1.286	0.224
TKI				
CEA	5	22	4.696	<0.001^a^
CYFRA 21-1	6	21	4.219	<0.001^a^
CA 19-9	5	20	0.656	0.519
CA 125	5	19	2.424	0.024^a^
NSE	6	16	1.492	0.151

a P<0.05. NO, surgery only; TP, paclitaxel or docetaxel; GP, gemcitabine; PP, pemetrexed; TKI, tyrosine kinase inhibitor; CEA, carcinoembryonic antigen; CYFRA 21-1, cytokeratin 19 fragment; CA, carbohydrate antigen; NSE, neuron-specific enolase; CR, complete response; PR, partial response; NC, no change; PD, progressive disease.

**Table V.** Prognostic profile of biomarkers for responses to chemotherapy.

Marker	AUC	Sensitivity (%)	Specificity (%)	95% CI	

				Lower	Upper
CEA	0.738	81.4	55.6	0.654	0.822
CYFRA 21-1	0.779	90.0	60.3	0.700	0.858
CA 19-9	0.610	62.9	54.0	0.514	0.705
CA 125	0.628	90.0	30.2	0.533	0.722
NSE	0.747	70.0	74.6	0.663	0.831

AUC, area under the curve; CEA, carcinoembryonic antigen; CYFRA 21-1, cytokeratin 19 fragment; CA, carbohydrate antigen; NSE, neuron-specific enolase.
